# Can microscopic ileitis in patients with clinically suspected inflammatory bowel disease predict the future?

**DOI:** 10.1186/s12876-020-01207-0

**Published:** 2020-03-05

**Authors:** Fadi Abu Baker, Jesus Alonso Z’cruz De La Garza, Smadar Nafrin, Amir Mari, Muhammed Suki, Baruch Ovadia, Oren Gal, Yael Kopelamn

**Affiliations:** 1 Department of Gastroenterology and Hepatology, Hillel Yaffe Medical Center (Affiliated to the Technion Faculty of Medicine, Haifa, Israel), Ha-Shalom St, 38100 Hadera, Israel; 2 Department of Surgery, Hillel Yaffe Medical Center (Affiliated to the Technion Faculty of Medicine, Haifa, Israel), Hadera, Israel; 3 Department of Gastroenterology, Nazareth EMMS Hospital (Affiliated with the Faculty of Medicine, Bar Illan University), Nazareth, Israel

**Keywords:** Ileocolonoscopy, Inflammatory bowel disease (IBD), Microscopic ileitis (MI), Terminal ileum

## Abstract

**Background:**

The implication of microscopic ileitis finding in patients referred for ileocolonoscopy for clinically suspected inflammatory bowel disease (IBD) is not well defined, and its correlation with clinical outcome has not been fully studied. The current study aims to determine the prognostic yield of biopsies in this setting, and to evaluate the correlation of microscopic ileitis with long-term clinical outcome.

**Methods:**

We reviewed endoscopic reports of patients referred to our department for ileocolonoscopy in the years 2010–2016, as part of a diagnostic work-up for suspected IBD. Patients whose ileocolonoscopies proved normal were included, provided that terminal ileum biopsies had been performed. Accordingly, patients were divided into groups classified as normal (normal or reactive changes) and microscopic ileitis (inflammation or ileitis of any severity). Both groups were followed prospectively to determine clinical outcome.

**Results:**

A total of 439 patients met the inclusion criteria. Sixty-four (14.6%) showed inflammation on biopsy and were included in the microscopic ileitis group. Age range and gender figures did not differ significantly between the groups. Overall follow-up period was 6.1 ± 2.3 years. Patients in the microscopic ileitis group were significantly associated with Crohn’s diagnosis during the follow-up period compared with the normal group (19% vs 2%, OR = 11.98, 95%CI = 4.48–32.01; *p* < 0.01). Patients with granuloma or moderate-severe ileitis on biopsy were significantly associated with Crohn’s development (100% vs 11%; *P* < 0.01) compared with mild or nonspecific inflammation.

**Conclusion:**

The discovery of microscopic ileitis in clinically suspected IBD is associated with increased risk of future diagnosis of Crohn’s disease.

## Background

Ileocolonoscopy that includes performance of terminal ileum biopsies plays an essential role in the diagnostic work-up for suspected inflammatory bowel disease (IBD). It may aid in ruling out various infectious, inflammatory or functional disorders that may mimic IBD [1–3]. When typical or suspicious endoscopic findings are encountered, multiple representative biopsies from terminal ileum (TI) and colon segments are warranted [4].

Performance of biopsies on a colon and a TI that appear normal during endoscopy, however, constitutes a dilemma for practitioners, as they are willing to complete the diagnostic evaluation by integrating histological data, but they are aware of the possible low diagnostic yield as well as the risks of prolonged or complicated procedures [5].

The importance of random biopsies in specific indications in a colon that appears normal in endoscopy has been proved in several studies [6]. However, the role of biopsies in the TI under these circumstances is still not well defined. The predominant attitude in the literature is that biopsies from a normally appearing TI have a low diagnostic yield and are not routinely recommended [7, 8].

However, the clinical setting and the indication for the procedure is of paramount importance and may be correlated with the diagnostic yield when performing biopsies in a suitable setting [9–11]. Thus, TI biopsies may be of greatest value for patients who undergo endoscopy for known or strongly suspected Crohn’s disease [12–14]. Moreover, cases have been demonstrated of inflammatory as well as infectious disorders that have been diagnosed merely by biopsy when the TI appeared normal in endoscopy [15, 16].

In our practice, during the ileocolonoscopy procedure in patients referred for suspected IBD, biopsies are usually undertaken from the TI whether or not it shows a normal endoscopic appearance. Histopathologic examinations of these biopsies frequently show normal findings, but frequently reveal changes that are consistent with chronic ileitis or other nonspecific inflammation. The significance of such microscopic ileitis (MI) and the correlation of these histological findings with long-term clinical outcome are unknown. These issues are addressed in this study and compared with the outcome of normal TI biopsy findings to appreciate the predictive value of MI in future diagnosis of Crohn’s disease.

## Methods

We searched endoscopy reports to identify all patients referred to the gastroenterology department of the Hillel-Yaffe Medical Center, a university-affiliated hospital in Israel, for ileocolonoscopies in the years 2010–2016 as part of diagnostic work-ups for suspected IBD. These patients suffered various clinical presentations such as chronic abdominal pain, diarrhoea and anal complaints deemed by the referring gastroenterologist to raise suspicions of IBD. Patients were included if their ileocolonoscopies were normal, provided that TI biopsies had been performed. Normal ileocolonoscopy denoted findings of normal colon and TI mucosa. Mild nodularity characteristic of lymphoid hyperplasia was also considered normal. Findings of erythema, friability, granularity, erosions, ulcers or strictures were considered abnormal and were not included in the study. Moreover, only patients whose data were complete and therefore included demographic details (age, sex), indication for exam, endoscopic findings, and the availability of biopsy results were included in the final analysis. Patients with prior colonoscopies or IBD diagnoses, as well as patients who had undergone prior colon resection, were excluded.

Our staff comprised six senior endoscopists (who had more than 15 years’ experience and who had performed more than 500 annual colonoscopies), who performed these ileo-colonoscopy procedures or directly supervised their performance by trainees to ensure visualisation and assessment of TI mucosa. More than 95% of the patients who were included in the study had undergone adequate bowel preparation (some patients were included after repeated exams due to poor preparation).

**Fig. 1:**
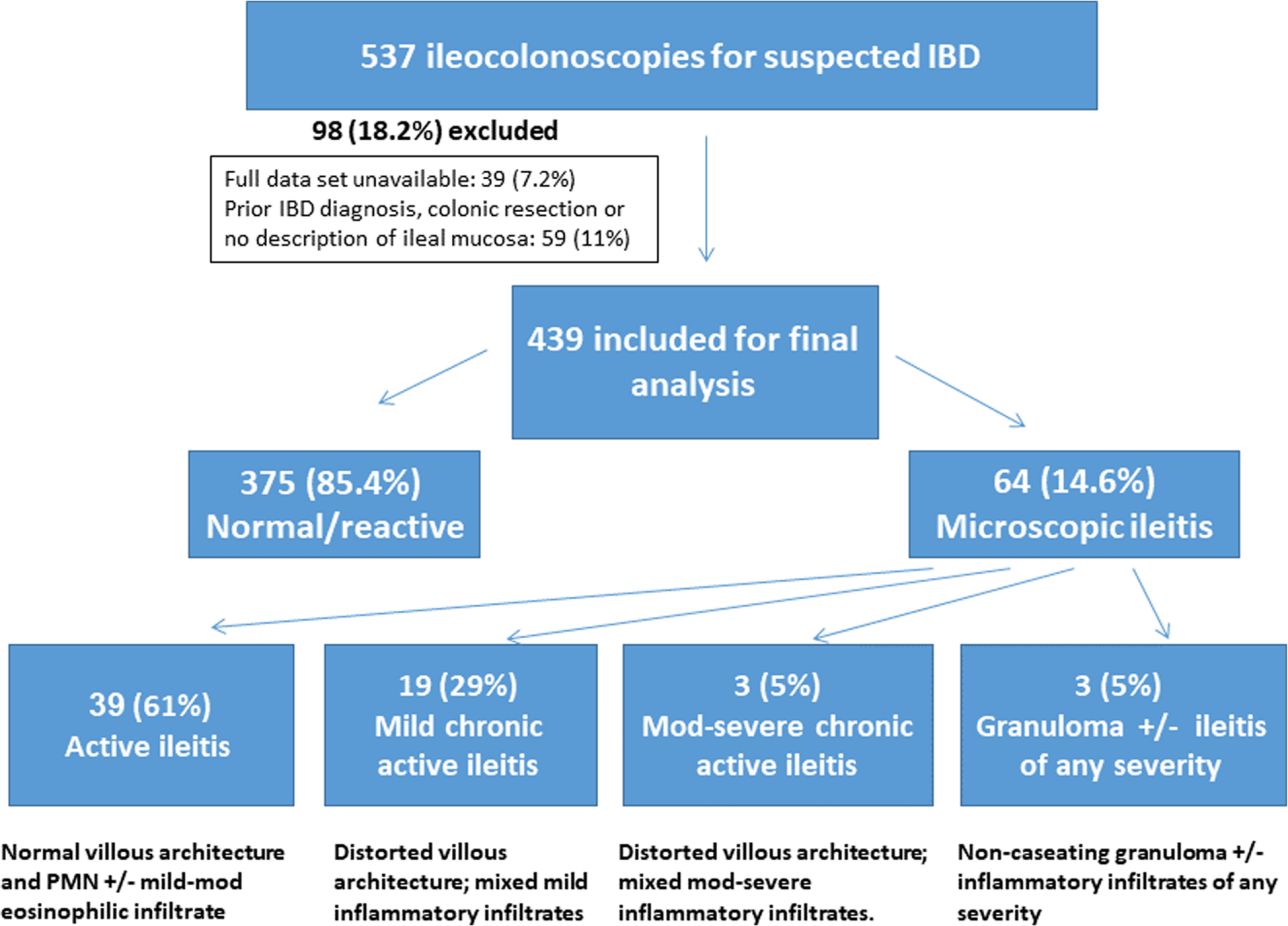
Study population and group definitions

Patients were placed into groups categorised as normal or MI according to the biopsy results (Fig. 1). The normal group included patients who showed normal biopsies or reactive and nonspecific changes, while the MI group included patients who were found to have active ileitis (normal villous architecture and polymorphonuclear or mild to moderate eosinophilic infiltrate), chronic active ileitis (distorted villous architecture; mixed inflammatory infiltrates of any severity) and granulomatous inflammation (non-caseating granuloma formation, no evidence of fungi or parasites). In order to determine the long-term clinical outcome of the patients in both groups, we reviewed electronic files of clinic, imaging and endoscopic reports available in our department. For patients who continued follow-up elsewhere, we used a national electronic system that enabled access to patients’ reports and clinical data, although access was usually restricted for all data resources. Some of these patients were invited for clinical assessment in our department. The follow-up was usually discontinued once a new diagnosis of Crohn’s disease was confirmed or with the last report available by the time this study had started.

A Crohn’s diagnosis was counted when an expert gastroenterologist clearly confirmed that the patient had undergone a subsequent follow up examination that included recurrent ileo-colonoscopy, capsule endoscopy and/or bowel-imaging procedures and new findings had been produced that met the diagnostic criteria for Crohn’s disease. We performed also multivariate analysis to identify independent association of age, sex and MI with Crohn’s disease development. We classified patients in the MI group according to inflammation severity in order to evaluate histological correlation with final outcome.

The local institutional Helsinki ethics board in Hillel Yaffe Medical Center approved the study (reference number 0112–17) and granted exemption from the requirement to obtain informed consent as patients were receiving standard care that did not relate to the study. Data collection did not influence medical practice.

### Statistical analysis

Continuous parameters were presented by means ±standard deviations, and categorical parameters were expressed by use of frequencies and percentages. Differences between the MI and normal groups were compared by t-test for quantitative parameters, and Fisher’s exact test for the categorical parameters. Multivariate analysis by logistic regression was used for prediction of subsequent Crohn’s disease diagnosis by age, gender and group. *P* < 0.05 was considered as significant. The software package SPSS version 25 was used for all statistical analyses.

## Results

Overall, the records and endoscopic reports regarding 537 patients who had undergone ileocolonoscopy for suspected IBD and whose findings were normal were reviewed. Thirty-nine (7.2%) patients did not have full data sets and were excluded. Fifty-nine (11%) patients had prior colonoscopy, IBD diagnosis or colonic resection and were excluded as well. A total of 439 patients were considered suitable and these patients were included in the final analysis. Of these, 64 (14.6%) had evidence of inflammation of any severity on biopsy and were included in the MI group, while 375 (85.4%) patients had unremarkable or reactive findings and were included in the normal biopsy group. Figures of average age (30.8 ± 15.6 vs 33.2 ± 16.6, *P* = 0.25) and male gender (48.5% vs 50%; *P* = 0.89) did not differ significantly between the groups. The follow-up period was 5.5 ± 2.3 and 6.7 ± 2.3 years for the MI and normal groups, respectively (Table 1). Sixty per cent of these patients were followed up by IBD experts in our department, 15% by IBD experts in other hospital departments while the others continued follow-up by referral to gastroenterologists in a health maintenance organisation setting.

**Table 1:** Baseline characteristics and patient outcomes in both study groups

Patients	Normal biopsy (*N* = 375)	Microscopic Ileitis (*N* = 64)	*P* Value
Age (years)	30.8 ± 15.6	33.2 ± 16.6	*P* = 0.25
Sex (male)	182 (48.5%)	32 (50%)	*P* = 0.89
Follow-up (years)	6.7 ± 2.3	5.5 ± 2.3	*P* = 0.08
Crohn’s diagnosis	7 (2%)	12 (19%)	***P*** **< 0.01**

Patients in the MI group were significantly associated with a subsequent Crohn’s disease diagnosis during the follow-up period as compared with the normal biopsy group (19% vs 2%; *p* < 0.01). In multivariate analysis, finding of MI were highly associated with later diagnosis of Crohn’s as compared with the normal biopsy group (OR = 11.98, 95%CI = 4.48–32.01; *P* < 0.01), while age (OR 0.99, 95%CI 0.96–1.02; *P* = 0.68) or gender (OR 1.77 95%CI 0.65–4.8; *P* = 0.26) were not. According to the available data, the majority of the patients developed mild-to-moderate, non-extensive and uncomplicated disease, and all of them received active treatment. Interestingly, the vast majority of the Crohn’s diagnoses were made within 3 years of the index ileocolonoscopy.

The histological findings for patients with MI are summarised in Table 2. The most frequent finding was active ileitis (61%) followed by mild active chronic ileitis (29%). Patients who exhibited moderate-severe ileitis or granuloma on the biopsy specimens were significantly associated with subsequent Crohn’s diagnosis as compared with mild or nonspecific inflammation (100% vs 11%; *P* < 0.01).

**Table 2:** Biopsy findings and their correlation with diagnosis of Crohn’s disease during follow-up in the microscopic ileitis group

Biopsy findings	Number of patients N (%)	Number of patients with Crohn’s diagnosis N (%)
Active ileitis	39 (61%)	2 (3.1%)
Mild chronic active ileitis	19 (29%)	4 (21%)
Moderate-severe chronic active ileitis	3 (5%)	3 (100%)
Granuloma +/− ileitis of any severity	3 (5%)	3 (100%)

## Discussion

The current study was designed to evaluate the significance of MI and its prediction value in the diagnosis of Crohn’s disease and to assess the diagnostic yield of TI biopsy in cases in which patients were clinically suspected to have IBD but who were categorised by endoscopy as normal.

Firstly, we demonstrated that the rate of discovery of abnormal biopsy results among these patients whose TIs appeared normal was 14.5%. This rate is clearly high when compared with reports from studies in which ileocolonoscopies were performed for other, different indications. Jonathan et al. retrospectively reviewed the cases of 414 consecutive patients who had undergone terminal ileal biopsies and who had been referred for various indications and found that, in cases in which the TI was grossly normal, only 5.1% of biopsies were histologically abnormal, and only 4.2% had significant histologic inflammation [17]. Similarly, Melton et al., in a large retrospective study, found that 5% of ileal biopsies obtained from patients who had displayed normal endoscopy had abnormal histopathological findings [18].

Secondly, findings of MI in biopsy specimens were significantly associated with subsequent diagnosis of Crohn’s disease during follow-up, as compared with findings of no MI (normal biopsy) (19% vs 2%; OR = 11.98, 95%CI = 4.48–32.01; *P* < 0.01). This association was even more significant when only those patients with evidence of chronic inflammation on biopsy were included, as 40% of these patients were diagnosed with Crohn’s during the follow-up period. Moreover, the correlation of histological findings with patients’ outcomes revealed that findings of active ileitis without a chronic component or architecture distortion did not differ significantly from those of patients with normal biopsy (5% vs 2%; *p* = 0.11). We found that in all patients with moderate-severe or granulomatous inflammation, a diagnosis of Crohn’s disease was established at the end of the follow-up period.

Straightforward conclusions and recommendations for MI management in this setting may be hampered by the small number of patients that was considered in the current study. However, these patients may benefit from further investigation with capsule endoscopy, abdominal imaging as well as close and continuous follow-up. Thus, histological findings may direct the clinician’s decision regarding the necessity of further investigations or follow-up and contribute to improved patient management.

To our knowledge this is the first study to assess histological findings in this setting and to evaluate their correlation with patients’ outcomes. Generally speaking, the significance of MI and its clinical correlation is unknown. A small study by Díaz [19] has linked microscopic ileitis with chronic diarrhoea. However, we could find very few studies in the literature that addressed MI, and hence prospective studies to evaluate different aspects and associations of MI are warranted.

One main limitation of our study was the inability to access full medical investigations that had been performed on these patients during their follow-up periods. Therefore, we could not discover the exact percentage of patients who had been referred for abdominal imaging and/or small bowel capsule in both groups. This might have had an impact on the rate of diagnosis of Crohn’s disease. However, these patients were managed by expert gastroenterologists in specific clinics and apparently received similar care.

## Conclusions

In the setting of normal ileocolonoscopy of patients clinically suspected of suffering from IBD, histological findings from TI biopsies may be predictive of the clinical outcome.

## Data Availability

The datasets used and/or analysed during the current study are available from the corresponding author on reasonable request.

## Abbreviations

IBD: Inflammatory bowel disease
MI: Microscopic ileitis
TI: Terminal ileum
